# Split-GFP Reassembly Assay: Strengths and Caveats from a Multiparametric Analysis

**DOI:** 10.3390/ijms232113167

**Published:** 2022-10-29

**Authors:** Christophe Bignon, Antoine Gruet, Sonia Longhi

**Affiliations:** Laboratoire Architecture et Fonction des Macromolécules Biologiques (AFMB), UMR 7257, Centre National de la Recherche Scientifique (CNRS), Aix-Marseille University, 163 Avenue de Luminy, Case 932, CEDEX 09, 13288 Marseille, France

**Keywords:** protein complementation assays, protein–protein interactions, intrinsically disordered proteins, bipartite reporters, fluorescence

## Abstract

The split-Green Fluorescent Protein (GFP) reassembly assay is a powerful approach to study protein–protein interactions (PPIs). In this assay, two proteins, respectively, fused to the first seven and the last four β-strands of GFP are co-expressed in *E. coli* where they can bind to each other, which reconstitutes the full-length GFP. Thus, the fluorescence of the bacteria co-expressing the two fusion proteins accounts for the interaction of the two proteins of interest. The first split-GFP reassembly assay was devised in the early 2000s in Regan’s lab. During the last ten years, we have been extensively using this assay to study the interactions of an intrinsically disordered protein (IDP) with two globular partners. Over that period, in addition to accumulating molecular information on the specific interactions under study, we progressively modified the original technique and tested various parameters. In those previous studies, however, we focused on the mechanistic insights provided by the approach, rather than on the method itself. Since methodological aspects deserve attention and the best bipartite reporter to study PPIs involving IDPs remains to be identified, we herein focus on technical aspects. To this end, we first revisit our previous modifications of the original method and then investigate the impact of a panel of additional parameters. The present study unveiled a few critical parameters that deserve consideration to avoid pitfalls and obtain reliable results.

## 1. Introduction

Intrinsically disordered proteins (IDPs), i.e., proteins lacking a defined and stable secondary and tertiary structure, are characterized by their ability to establish a broad and complex partnership [1]. The description of IDP interactomes can be tackled both in silico [2] and experimentally [3,4]. Protein–protein interactions (PPIs) involving IDPs can be investigated through a broad range of biophysical and biological methods (reviewed in [5,6]). Among the latter, several protein complementation assays (PCAs) have been set-up (reviewed in [7]) which all share three common properties: first, a protein reporter is split into two halves (hereafter referred to as rep1 and rep2) that can reassemble in vivo or in vitro to reconstitute the native protein with all its characteristics; secondly, neither rep1 nor rep2 is endowed with the properties of the full-length reporter; thirdly, rep1 and rep2 do not reassemble spontaneously but only when they are, respectively, fused to each of two proteins (herein referred to as X and Y) whose interaction is being investigated. The third feature can either arise naturally [8,9] or be achieved by mutating the two halves until the desired low affinity is reached [10]. Note that in the former case, the spontaneous low rep1/rep2 reassembly is at least in part accounted for by the low solubility of each half considered individually. This leads to their precipitation in the cell expressing them if they are not timely drawn to each other by their fusion partners X and Y. 

In addition to being able to recover its native three-dimensional structure upon reassembly, a reporter is also selected upon its ability to provide an easy way to detect and even quantify the reassembly. For that reason, reporters emitting fluorescence (such as the green fluorescent protein (GFP)) or luminescence (the luciferase and its derivatives) are particularly sought after because assays using them are sensitive, inexpensive, and easy to set-up. Split-reporter complementation assays can be performed in vivo or in vitro. For in vitro assays, X-rep1 and Y-rep2 fusion proteins are expressed separately and purified before testing their ability to reassemble [11,12]. This additional step is not required for in vivo assays, of which split-GFP reassembly assay is the archetype.

**Figure 1:**
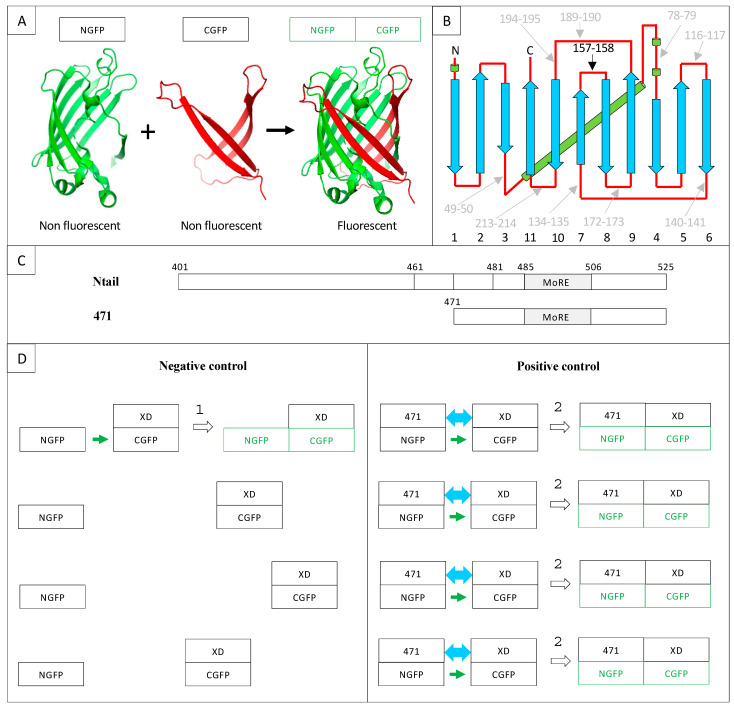
(**A**) Split-GFP reassociation. Cartoon representation illustrating how NGFP (1–157) and CGFP (158–238) reassociate to recreate the fluorescent protein. (**B**) GFP topology diagram. β-strands are represented as blue arrows and numbered below the drawing, α-helices are represented as green cylinders and loops as red lines. The internal α-helix containing the chromophore is represented behind the structure. N, N-terminus. C, C-terminus. The different splitting points tested by Abedi et al. [19] are located on the GFP structure. Cutting sites resulting in low GFP fluorescence are in grey, while the cutting point eventually retained by Ghosh et al. [13] (i.e., 157–158) is shown in black. (**C**) Full-length Ntail and its truncation variant 471. Numbers refer to amino acid numbering of the full-length nucleoprotein. The molecular recognition element (MoRE, 485–506), which interacts with XD, is indicated. The N-terminus of truncation variants 461 (461–525) and 481 (481–525) used in Section 2.8 are also located on the full-length sequence. The truncation variant 471 (471–525) used as positive control in this study is represented below the full-length sequence. (**D**) Negative and positive controls of 471/XD interaction in the split-GFP reassembly assay. NGFP is the N-terminal half of GFP (residues 1 to 157, **A**); CGFP is the C-terminal half of GFP (residues 158–238, **A**); NGFP-471 is the fusion of the NGFP half of GFP with 471; XD-CGFP is the fusion between XD and the CGFP half of GFP. The green color indicates that the reassociation of the two GFP halves results in a fluorescent protein (**A**). For the sake of clarity, only four potentially interacting molecules are represented in the following examples. (1) Negative control: since only XD is fused to CGFP, the interaction between the two GFP halves is not promoted by the high-affinity interaction between 471 and XD as it is in the positive control. Therefore, the probability that one molecule of NGFP (which is referred to as “stop” throughout the text) interacts with one molecule of XD-CGFP via the sole NGFP/CGFP interaction (small green arrow) is low, which leads to low fluorescence background (symbolized by the formation of only one NGFP/XD-CGFP complex in the Figure). (2) Positive control: because of the high affinity between 471 and XD, the probability that one molecule of NGFP-471 fusion protein encounters one molecule of XD-CGFP fusion protein (blue double arrow) is much higher than in the negative control, which produces more fluorescence than the negative control (symbolized by the formation of four NGFP-471/XD-CGFP complexes in the Figure).

GFP is a β-barrel made of 11 β-strands linked by loops. As a consequence, all the loops are clustered at the “top” and “bottom” of the barrel (Figure 1A,B). 

The first split-GFP reassembly assay was a bipartite assay developed in the early 2000s [8,13]. The reporter used was sg100, a brighter variant (F64L, S65C, Q80R, Y151L, I167T, K238N) than *wild-type* GFP (wtGFP) with a single excitation peak. Yet, some bona fide interactions escaped detection, and sg100 was thereafter replaced with a “folding reporter” GFP (frGFP) (F64L S65T, F99S, M153T, V163A [14]) providing stronger fluorescent signals than sg100 [15]. Eventually, further improvement was obtained by Blakeley et al. [16] who devised a “super positive” GFP (spGFP) featuring a much higher net charge (+34) than that of sg100 (–8), based on the observation that increasing protein charge is associated with increased solubility. In addition to sg100, frGFP and spGFP, split enhanced GFP (eGFP [17]) and split eYFP [18] have also been used. In the case of sg100, fr and spGFP, the bipartite reporter was generated by cutting the loop linking β-strands 7 and 8 between residues 157 and 158, which yields two moieties, respectively made of the first seven (NGFP) and the last four (CGFP) β-strands. This choice stemmed from circular permutation experiments that had identified this cutting point as the least destabilizing for GFP structure and function when a peptide was inserted between residues 157 and 158 [19] (Figure 1A,B). As mentioned above, NGFP or CGFP derived from sg100 are characterized by a drastically reduced solubility compared to full-length sg100. Accordingly, they are prone to precipitate when expressed in *E. coli*. However, fusing two interacting X and Y proteins in an anti-parallel configuration [13], respectively, to the C-terminal end of NGFP and to the N-terminal end of CGFP gives NGFP and CGFP a chance to evade precipitation. Indeed, when NGFP-X and Y-CGFP fusions are co-expressed in *E. coli*, X and Y bind to each other within the cell, which allows NGFP and CGFP to reconstitute the soluble and fluorescent GFP. Thus, there is competition between two concomitant events: the spontaneous tendency of NGFP and CGFP to precipitate and the ability of their fusion partners X and Y to allow them to avoid precipitating by interacting. As a result, the efficiency of the NGFP/CGFP reassembly process is dependent on the encounter probability of NGFP-X and Y-CGFP within the cell. In this respect, X and Y pairs consisting of small proteins endowed with high soluble expression capabilities in vivo and high reciprocal affinity will provide the highest probability of GFP reassembly. In addition, increasing the folding efficiency (frGFP) and/or decreasing the aggregation propensity (spGFP) of the GFP reporter will also increase the fluorescent signal. 

The irreversible nature of GFP reassembly allows accumulating reconstituted fluorescent molecules during the whole protein-expression period, which can extend up to 96 h, thereby enabling the detection of even weak and/or transient interactions. The cumulative fluorescent signal available at the end of the experiment is further increased by using GFP variants that are brighter than wtGFP. Due to their biased amino acids composition toward polar and charged residues, IDPs are generally easier to express soluble than globular proteins in *E. coli* [20]. Perhaps unsurprisingly, the bipartite split-GFP complementation assay was historically set-up for studying the interaction of two peptides [8,13] with a very high predicted solubility that, in addition, proved to be expressed at a high level in *E. coli* [21]. 

During the last decade, we have extensively used this split-GFP reassembly assay to investigate the interactions between the intrinsically disordered C-terminal domain of the measles virus nucleoprotein (Ntail) [22] and two of its natural partners, namely the C-terminal X domain (XD) of the viral phosphoprotein [23,24] and the major inducible 70 kDa heat shock protein (HSP70) [25,26,27]. The approach turned out to be well suited to achieve insights into the underlying molecular mechanisms governing Ntail interaction with XD and HSP70 [28,29,30,31]. In those studies, we focused on the mechanistic insights provided by the approach, rather than on the method itself, with technical details only being mentioned in the Materials and Methods and/or in supplementary material. 

Methodological aspects, however, deserve attention to avoid possible pitfalls. This is of even further relevance considering that split-GFP reassembly is a broadly used technique and that the best bipartite reporter to study IDP interactions remains to be identified. With this in mind, and based on our experience gathered over years, in the present manuscript we have decided to focus on methodological aspects and discuss potential traps that are inherent to specific features of the method. To this end, we first revisit our previous modifications of the original method and then investigate the impact of a panel of additional parameters, including culture medium supplier, GFP variants, the presence of a hexahistidine tag, the use of Gateway vectors, the interaction strength of the partner proteins and the choice of the directionality of the fusion construct, in order to identify possible experimental conditions enabling the even better exploitation of this powerful approach to document PPIs involving IDPs and to shed light on their molecular mechanisms. We mainly conducted this multiparametric analysis in the context of the Ntail truncated variant 471 (Figure 1) because it yields higher fluorescence values than full-length Ntail whatever the interaction partner [29]. Figure 1 also provides a scheme of the interaction between this Ntail truncation variant and XD in the context of the split-GFP reassembly assay.

## 2. Results and Discussion

### 2.1. Experimental Conditions

With respect to the method described in the original publication [8], our first modification was to measure the fluorescence of bacteria using 96-well clear bottom black plates and a microplate reader, rather than to estimate by visual inspection the fluorescence produced by colonies on plate. This allows running each experimental point in triplicate for a more reliable estimation of fluorescence intensity and of cell density at the end of the culture so that normalized and quantitative results can be generated [21]. 

We also ran an experiment aimed at optimizing expression conditions. This was achieved by using a fractional factorial approach (full factorial = 324) comprising 24 combinations of three temperatures (37 °C, 25 °C, 17 °C); three culture media (LB, TB, 2YT); four *E. coli* strains (BL21 pLysS, Rosetta pLysS, T7 pRos, C41 pRos); three IPTG concentrations (0.01 mM, 0.1 mM, 0.5 mM); and three arabinose concentrations (0.02%, 0.2%, 2%) [21,32]. Surprisingly, the best combination (17 °C, TB, T7 pRos, 0.5 mM IPTG, 2% arabinose) determined by performing a trend analysis, as described in Benoit et al. [32], appeared to be very different from that used in the original publication [8]. The same was true for both the proteins studied in our lab (Ntail/XD) and for the proteins used by Magliery et al. [8] (Leucin zippers), with a signal-to-noise ratio (SNR) increase from 3.7 to 6.4 for the Ntail/XD pair and from 14.5 to 17.3 for leucin zippers [21], suggesting that these expression conditions are not protein-dependent but may be generally applicable.

### 2.2. Effect of Culture Medium Supplier

**Figure 2:**
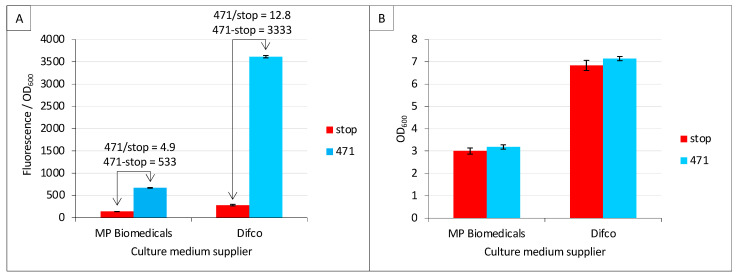
Culture medium brand is critical. Split-GFP reassembly assay was performed as described in Materials and Methods using negative (stop, in red) and positive (471, in blue) controls of the interaction between 471 and XD. (**A**) Fluorescence-to-OD600 ratio (mean value and standard deviation of a triplicate experiment). The ratio and the difference of the positive to negative control are indicated above the histogram. (**B**) OD_600_ at the end of the culture of the same samples. The name of the TB suppliers is indicated on the x-axis.

Among the new parameters tested in the present study, we first addressed the question as to whether the culture medium supplier may have an impact on fluorescence values. To this end, we tested two different suppliers of TB medium, namely Difco and MP Biomedicals. Difco provided a higher fluorescence-to-OD_600_ ratio (stop_Difco/stop_MP Biomedicals = 2.1; 471_Difco/471_MP Biomedicals = 5.4) (Figure 2A). Interestingly, the increased background (stop_Difco/stop_MP Biomedicals = 2.1) observed in Difco TB did not result in lower SNR (471/stop) compared to MP Biomedicals TB but led to the opposite result (12.8 versus 4.9). The latter result is even more impressive when data are expressed as a difference (471—stop), leading to 3333 versus 533. Since a lower fluorescence-to-OD_600_ ratio could be due to higher biomass (higher OD_600_), the mean and standard deviation of the latter were measured and reported in Figure 2B. The results revealed the opposite scenario, where the biomass at the end of the culture was lower with MP Biomedical TB than with Difco TB, and this was independent of the expression construct (i.e., stop and 471 provided identical results). The data reported in Figure 2 can be collectively described as follows: the TB medium providing the lowest final biomass also provided the lowest fluorescence to OD_600_ ratio. This means that the brand of TB has an effect on both cell growth (Figure 2B) and the amount of fluorescence generated by each cell (Figure 2A). The composition of the two TBs is similar. Both use 12 g/L of pancreatic digest of casein (tryptone), 24 g/L of yeast extract and 4 mL/L of glycerol. However, they differ in the salt concentration: Difco TB uses 9.4 g/L of dipotassium phosphate and 2.2 g/L of monopotassium phosphate, whereas MP Biochemical TB contains 12.5 g/L of dipotassium phosphate and 2.3 g/L of monopotassium phosphate.

### 2.3. Impact of GFP Variants

**Figure 3:**
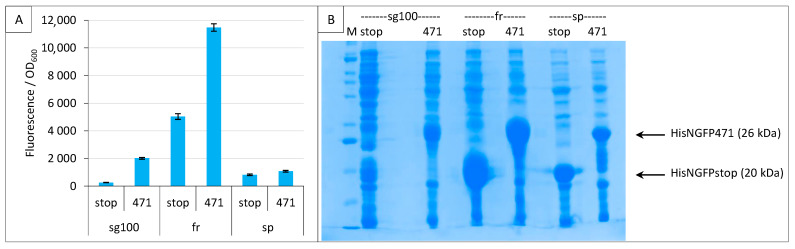
Comparison of sg100, fr and sp GFP variants at a single temperature. Split-GFP reassembly assay results as obtained with the negative (stop) and positive (471) controls of the 471/XD interaction with sg100, fr and sp GFP reporters at 17 °C. (**A**) Fluorescence to OD_600_ ratios. (**B**) SDS-PAGE analysis of the expression of the NGFP fusions. M, molecular size markers (200, 150, 100, 85, 60, 50, 40, 30, 25, 20, 15, 10 kDa).

We then compared three GFP variants sg100 [8], folding reporter (fr, [15]) and super positive GFP (sp, [16]) under the conditions defined in the previous two paragraphs. The folding reporter, fr, provided the highest fluorescence, with values being almost six times higher than sg100 (Figure 3A). However, the background was also high and although sg100 provided a lower signal, it provided a better SNR (7.8 for sg100 versus 2.3 for fr). The fluorescence obtained with sp was the lowest. An analysis on gel indicated that even NGFP fusions providing the lowest fluorescence values (sp) were expressed at detectable levels (Figure 3B).

**Figure 4:**
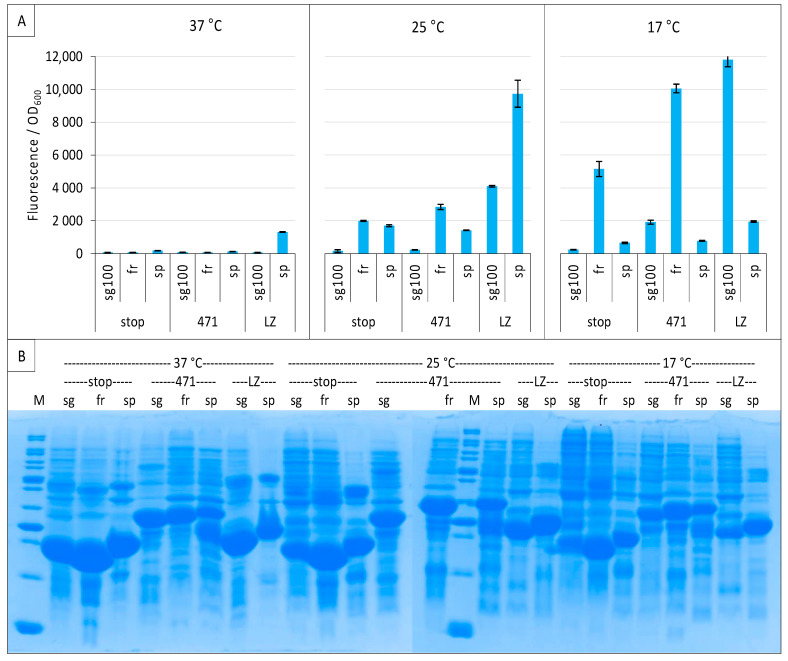
Comparison of sg100, fr and sp GFP variants at different temperatures. Split-GFP reassembly assay results as obtained with the negative (stop) and positive (471) controls of the 471/XD interaction with sg100, fr and sp GFP reporters at 37 °C, 25 °C and 17 °C. The interaction between leucine zippers (LZ) is included for sg100 and sp. (**A**) Fluorescence to OD_600_ ratios. (**B**) SDS-PAGE analysis of the expression of the NGFP fusions. M, molecular size markers (200, 150, 100, 85, 70, 60, 50, 40, 30, 25, 20, 15, 10 kDa).

Considering that the three variants could have different optimal temperatures, we performed the experiment again at 37 °C, 25 °C and 17 °C, and included leucine zippers as a positive control for sg100 [8,21] and spGFP [16]. Both sg100 and fr benefited from a decreased temperature, with 17 °C providing the best results (Figure 4A). By contrast, spGFP was most effective at 25 °C whatever the considered protein pair. Nevertheless, spGFP provided less fluorescence at its optimal temperature than fr for the 471/XD pair and sg100 for leucine zippers when these two reporters were used at their optimal temperature of 17 °C. Worse, the SNR obtained with spGFP at its optimal temperature (25° C) for the 471/XD pair was below 1 (0.84), suggesting that the signal is non-specific, contrary to the fluorescence obtained with fr (SNR = 1.42) and sg100 (SNR = 1.43) under the same experimental conditions. Thus, although it is derived from the super folder variant (see below), spGFP did not provide better results than the historical sg100 variant, at least when used with the proteins tested in our study. In this regard, it should be noted that spGFP is characterized by a high positive net charge (+34) while sg100 has a negative net charge (−8). The negatively charged 471 (−8.7 at pH 7.4) could not necessarily be the best fusion partner of super positively charged GFP fragments, which could explain the results that we obtained with this GFP variant. Figure 4B indicates that very low fluorescence intensities obtained at 37 °C were not due to a lack of expression of NGFP fusions. Incidentally, these experiments also revealed that His-NspGFP fusions migrate slower than their counterparts based on the other two GFP variants, presumably because of the charge difference. 

**Figure 5:**
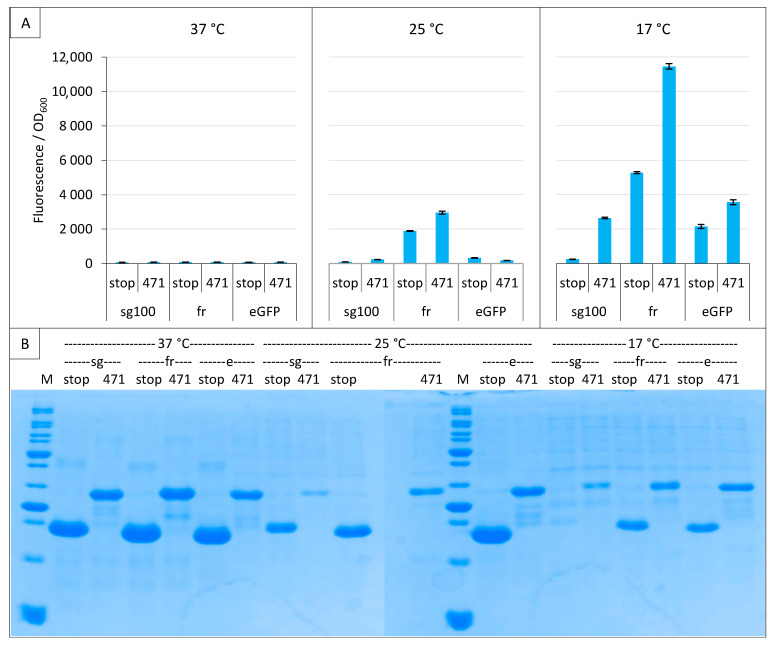
Comparison of sg100, fr and eGFP variants. Split-GFP reassembly assay results as obtained with the negative (stop) and positive (471) controls of the 471/XD interaction with sg100, fr and eGFP reporters at 37 °C, 25 °C and 17 °C. (**A**) Fluorescence to OD_600_ ratios. (**B**) SDS-PAGE analysis of the expression of the NGFP fusions. M, molecular size markers (200, 150, 100, 85, 70, 60, 50, 40, 30, 25, 20, 15, 10 kDa).

We next tested the enhanced GFP (eGFP). Figure 5A shows that eGFP optimal temperature is also 17 °C, but even at that temperature eGFP did not reach the fluorescence signal of fr nor the SNR of sg100 (10.6 for sg100 versus 1.6 for eGFP). Figure 5B shows that NGFP fusions were well expressed even at 37 °C where very low fluorescence values were observed.

**Figure 6:**
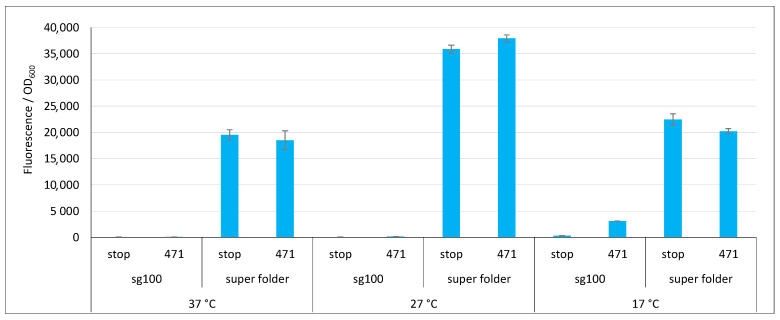
Comparison of sg100 and sf GFP variants. Fluorescence to OD_600_ ratios of a split-GFP reassembly assay as obtained with the negative (stop) and positive (471) controls of the 471/XD interaction with sg100 and sf GFP reporters at 37 °C, 27 °C and 17 °C.

Finally, we tested the super folder (sf, [33]). Figure 6 shows that, compared to sg100, sf provided very high fluorescence values at all three temperatures (with a maximum at 27 °C) and did so whatever the fusion partner. Despite these very high values, sf gave rise to a background that was equal to the signal. The most likely explanation is that NGFP and CGFP halves reassemble faster than 471 binds to XD. Thus, the reassembly of sf is not any more driven by the 471/XD interaction but by that of the two GFP halves. Although this feature prevents studying the binding of two proteins, it endows the system with potentially interesting applications. For instance, it can help the crystallization of a complex of two proteins interacting with a low affinity by maintaining them close to each other. However, this feature disqualifies sf as a reporter in a split-GFP reassembly assay since it does not comply to the third requirement described for rep1 and rep2 in the introduction. On the basis of these results, only sg100 and fr variants were further studied.

### 2.4. Addition of a Histidine-Tag to CGFP and Ensuing Analysis of CGFP Stability

**Figure 7:**
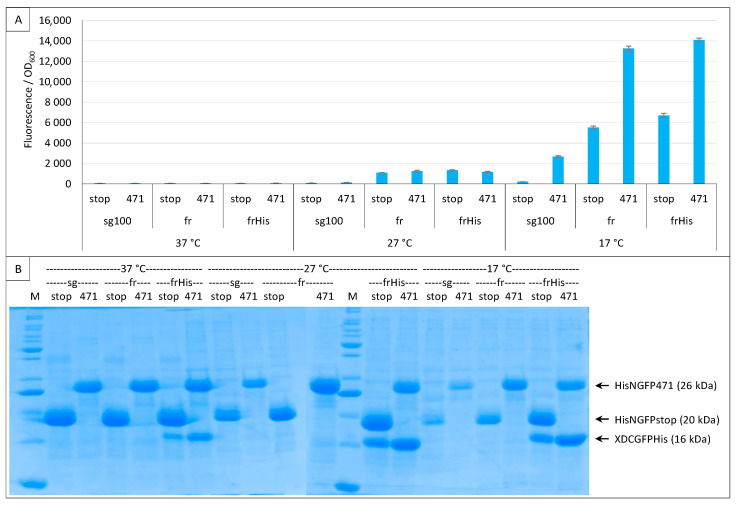
His-tagging CGFP fusion. Split-GFP reassembly assay results as obtained with the negative (stop) and positive (471) controls of the 417/XD interaction with sg100 and fr GFP reporters at 37 °C, 27 °C and 17 °C. In the case of fr, results obtained using His-tagged (frHis) and non-His-tagged (fr) XD-CfrGFP are shown. (**A**) Fluorescence to OD_600_ ratios. (**B**) SDS-PAGE analysis of the expression of the NGFP and CGFP fusions. M, molecular size markers (200, 150, 100, 85, 70, 60, 50, 40, 30, 25, 20, 15, 10 kDa).

The above experiments were performed following the original protocol that relies on a non-tagged CGFP. Under these conditions, the expression/stability of CGFP halves alone cannot be checked by SDS-PAGE. Therefore, we added a His-tag at the C-terminal end of the frCGFP fusion (XD-CfrGFP-His) and tested the His- and non-His-tagged XD-CfrGFP by a split-GFP reassembly assay. Figure 7A shows that adding a His-tag at the C-terminal end of XD-frCGFP does not change the fluorescence results, and Figure 7B that XD-CfrGFP-His could be easily detected on gel after IMAC purification onto a Ni column.

Figure 7B also highlights a surprising result: at 17 °C, the difference in the amount of XD-CfrGFP-His between the negative (stop) and positive (471) controls mirrors the fluorescence values (SNR = 2.1), suggesting that only the fraction of XD-CfrGFP-His that reassembled with its NfrGFP counterpart is protected from degradation and, hence, is involved in the production of fluorescence. However, this was not true at 27 °C (SNR = 0.9) nor at 37 °C (SNR = 1), temperatures at which the same difference in the amount of XD-CfrGFP-His could be detected by SDS-PAGE.

**Figure 8:**
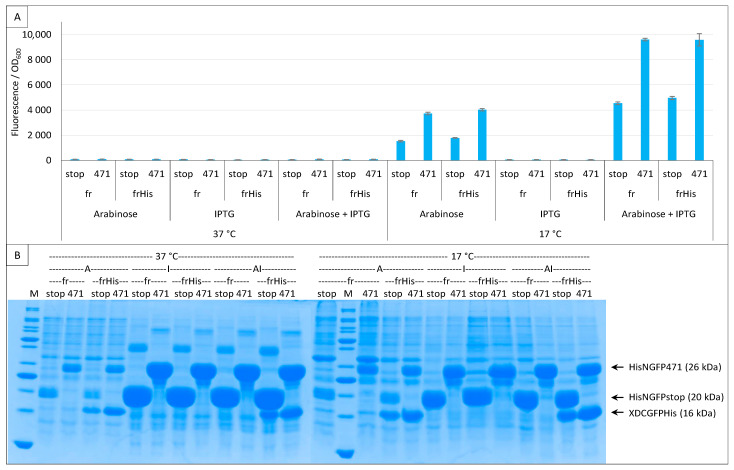
Single and double induction of expression. Split-GFP reassembly assay results as obtained with different inducer combinations and with the negative (stop) and positive (471) controls of the 471/XD interaction with the fr GFP reporter at 37 °C and 17 °C. Results obtained using His-tagged (frHis) and non-His-tagged (fr) XD-CfrGFP are shown. (**A**) Fluorescence to OD_600_ ratios. Inducers and inducer combinations are indicated on the x-axis. (**B**) SDS-PAGE analysis of the expression of the NGFP and CGFP fusions. A, arabinose; I, IPTG; AI, arabinose plus IPTG. M, molecular size markers (200, 150, 100, 85, 70, 60, 50, 40, 30, 25, 20, 15, 10 kDa).

To assess whether the observed differences in the amount of XD-CfrGFP-His between negative (stop) and positive (471) controls were conditioned by the co-expression of N- and C-GFP fusions, we performed the same experiment but used the two expression inducers arabinose and IPTG either separately or in combination. The results (Figure 8A) revealed that pET11a is a leaky vector expressing basal levels of His-NfrGFP fusions in the absence of IPTG (i.e., condition “Arabinose” in Figure 8A), whatever the expression temperature. 

The expression leakage of the vector encoding His-NfrGFP fusions precluded the possibility of ascertaining if the difference in the amount of XD-CfrGFP-His between negative (stop) and positive (471) controls persists in the absence of NfrGFP expression. Figure 8B confirms that, in the presence of arabinose only (CGFP fusion inducer), the bands corresponding to His-NfrGFP-stop and His-NfrGFP-471 are also detectable at 37 °C and 17 °C, although this co-expression only led to detectable fluorescence values at the optimal temperature of 17 °C. By contrast, the addition of IPTG only (NGFP fusion inducer) resulted in a significant increase in the amount of both His-NfrGFP-stop and His-NfrGFP-471 but in no detectable expression of XD-CfrGFP-His whatever the temperature (Figure 8B), which is in line with the observed absence of fluorescence when this inducer was used alone (Figure 8A).

**Figure 9:**
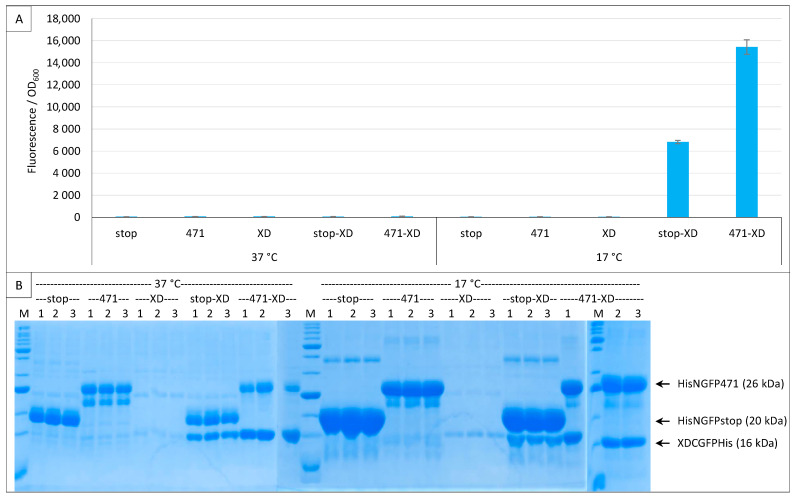
XD-CGFP is degraded when expressed alone. Split-GFP reassembly assay results as obtained with different combinations of plasmids and with the negative (stop) and positive (471) controls of the 471/XD interaction with the fr GFP reporter at 37 °C and 17 °C. Only pMRBad encoding XD-CfrGFP-His was used. (**A**) Fluorescence to OD_600_ ratios. All experimental points were induced with both arabinose and IPTG. Fusion proteins (stop, 471, XD) and fusion protein combinations (stop-XD, 471-XD) are indicated on the x-axis. (**B**) SDS-PAGE analysis of the expression of the NGFP and CGFP fusions. Triplicates were loaded individually (1, 2, 3). M, molecular size markers (200, 150, 100, 85, 70, 60, 50, 40, 30, 25, 20, 15, 10 kDa).

To overcome the pET11a expression leakage issue, we transformed cells with either one or the two plasmids of the assay and used both inducers (arabinose + IPTG) in all experimental points. In this experiment, only fr was used because the His-tagged version of XD-CGFP did not exist for sg100. As expected, both plasmids were required to elicit fluorescence, and at 17 °C only (Figure 9A). 

Interestingly, XD-CfrGFP-His was barely detectable on gel when expressed alone (Figure 9B). The small amounts of XD-CfrGFP-His detected when expressed alone cannot be ascribed to a lack of expression because the protein could be readily detected on gel when co-expressed with His-NfrGFP-stop or His-NGFP-471, and in comparable amounts at 37 °C and 17 °C (Figure 9B). We conclude from this single plasmid experiment that XD-CfrGFP-His is well expressed at 37 °C and 17 °C, but it is degraded when it cannot reassociate with NfrGFP, irrespective of whether the latter is fused to 471 or not. Thus, it seems that it is the reassociation of the two GFP halves and not the binding of 471 with XD that prevents XD-CfrGFP-His from being degraded in vivo. However, since the two GFP halves reassociate faster when NfrGFP is fused to 471 than when it is expressed alone, it is logical that the positive control His-NfrGFP-471 provides more fluorescence than the negative control His-NfrGFP-stop, and that more XD-CfrGFP-His is found in gel lanes corresponding to the positive control His-NfrGFP-471 than in gel lanes corresponding to the negative control His-NfrGFP-stop because more XD-CfrGFP-His is protected per time unit.

However, we are unable to explain why no fluorescence was detected at 37 °C and 27 °C considering that all N- and C-GFP fusions are well expressed at both temperatures and that N- and C-GFP complexes form at all three temperatures (Figure 7B, Figure 8B and Figure 9B). One possible explanation comes from experiments using full-length GFP. When we expressed proteins fused to full-length eGFP, the fluorescence of bacteria increased as the temperature decreased with a maximum at 17 °C and a minimum at 37 °C (Bignon C., unpublished results), which suggests that the effect of temperature might be related to the reporter used (sf is almost insensitive to the temperature (Figure 6)) rather than to the fact that it is split. Taken together, our results suggest that reassembly complexes form at temperatures higher than 17 °C, but for unknown reasons do not reach full functionality at those temperatures to fluoresce (except for sf), although the reassembly does protect CGFP fusion partners from degradation. It is possible that for fr (and also presumably for sg100), a very low temperature such as 17 °C is mandatory for the formation of the chromophore, which is the last step of the folding process and takes about 4 h at 22 °C [34], whereas previous steps of the folding leading to the reassembly complex formation tolerate higher temperatures. That 17 °C proved to be the optimal temperature for sg100, fr and eGFP but not for sf and spGFP (Figure 3, Figure 4, Figure 5, Figure 6, Figure 7, Figure 8 and Figure 9) is in agreement with this hypothesis, since sp is derived from sf and sf has been evolved to fold and fluoresce more robustly than its fr ancestor [33]. Thus, our results might be GFP reporter-dependent and not a general feature of the split-GFP reassembly per se.

On the basis of these results and although Magliery et al. [8] reported that appending a His-tag at the C-terminal end of Csg100GFP prevented GFP reassembly, we also added a His-tag at the C-terminal end of Csg100GFP, which also proved effective (see Section 2.7).

### 2.5. Generation of a Gateway Vector for NGFP Fusions

**Figure 10:**
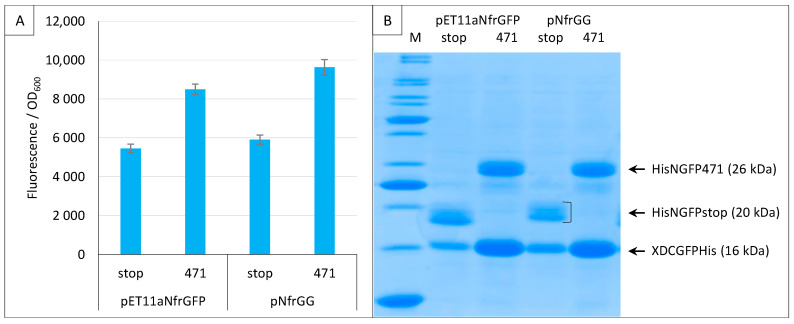
Gateway vector for expressing NfrGFP fusions. Split-GFP reassembly assay results as obtained with the Gateway and non-Gateway versions of plasmids expressing NfrGFP fusions and with the negative (stop) and positive (471) controls of the 471/XD interaction with the fr GFP reporter at 17 °C. Only pMRBad encoding XD-CfrGFP-His was used. (**A**) Fluorescence to OD_600_ ratios. (**B**) SDS-PAGE analysis of the expression of the NGFP and CGFP fusions. M, molecular size markers (200, 150, 100, 85, 70, 60, 50, 40, 30, 25, 20, 15, 10 kDa).

When we applied the split-GFP reassembly assay to the study of Ntail interactions, we modified the original pET11aNsg100GFP vector by replacing the multiple cloning site with a Gateway cassette [21]. Since all the experiments performed using the sg100 variant made use of this Gateway vector (pNGG), here, we also generated a Gateway version of pET11aNfrGFP (pNfrGG) and showed that replacing the restriction cloning sites with a Gateway cassette does not change the functional features of the plasmid (Figure 10).

### 2.6. GFP Variants and Detection of Low-Affinity Interactions

The experiments described above used a pair of proteins known to interact with a K_D_ in the µM range (471/XD) [35,36]. We previously showed that sg100 could be used to detect protein interactions with a lower affinity such as that existing between 471 and the human heat shock protein HSP70 [25,29]. Although fr produces a high fluorescence background, it also produces a much higher signal than sg100 (Figure 3). Therefore, we wondered whether the higher signal provided by fr could afford an advantage and, hence, be further exploited to study low-affinity protein interactions.

**Figure 11:**
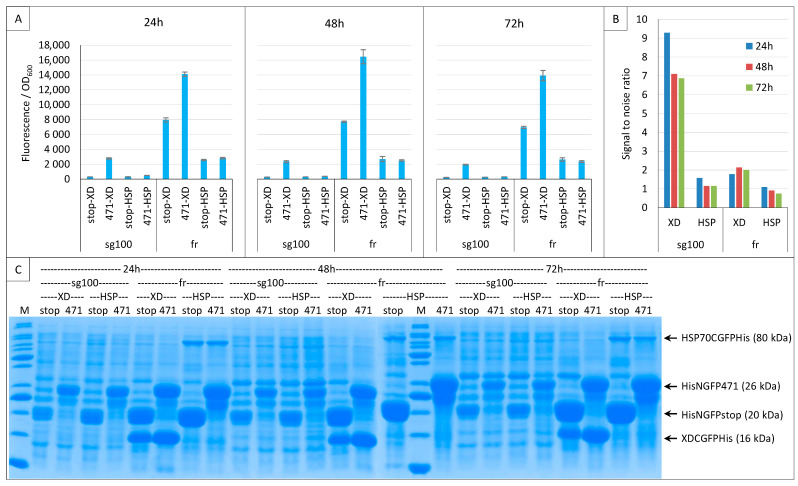
Low-affinity interactions. Split-GFP reassembly assay results as obtained at 24, 48 and 72 h with sg100 and fr GFP reporters and with negative (stop) and positive (471) controls of the 471/XD interactions at 17 °C. The sg100 experiments used non-His-tagged CGFP fusions, whereas fr experiments used His-tagged CGFP fusions. (**A**) Fluorescence to OD_600_ ratios. NGFP (stop, 471) and CGFP (XD, HSP) combinations are indicated on the x-axis. (**B**) Signal-to-noise ratio of the data in A. (**C**) SDS-PAGE analysis of the expression of the NGFP and CGFP fusions. M, molecular size markers (200, 150, 100, 85, 70, 60, 50, 40, 30, 25, 20, 15, 10 kDa).

To answer that query, we compared the results obtained with the two GFP variants while assessing the interaction of the 471/XD and 471/HSP70 bait–prey pairs. The experiment was only performed at 17 °C because our previous results indicated it was the optimal temperature for both variants, and it was run for three days. Almost identical results were obtained at 24, 48 and 72 h (Figure 11A) and, indeed, the signal obtained with 471/HSP70 was notably higher when fr was used instead of sg100. However, the background was also very high.

For an easier comparison, the SNR values of these experiments are reported in Figure 11B. This comparison indicates that sg100 always provided SNRs higher than 1 (reflecting a signal higher than the background) even in the case of the low-affinity 471/HSP70 interaction, whereas fr provided much lower SNRs for 471/XD and even SNR values below 1 in the case of 471/HSP70. Note that, on average, the highest SNRs were obtained after 24 h of culture. Thus, although fr provided much higher fluorescence signals than sg100 (Figure 11A), its low SNR renders it unsuitable for reliably detecting low-affinity interactions. Figure 11C indicates that sg100 provided higher SNRs than fr with lower steady-state levels of NGFP fusions.

To ascertain whether sg100 is a better reporter for low-affinity interactions, the same experiment was performed again using only 471 and HSP70 (Supplementary Figure S1). In all cases, the SNR was found to decrease from 24 to 72 h, but it always remained above 1 in the case of sg100, whereas for fr it was barely above 1 at 24 h and dropped below 1 at 48 and 72 h.

In conclusion, fr provided higher fluorescence values than sg100 by virtue of its intrinsic higher folding/reassembling rate and probably also by sustaining higher steady-state levels of NGFP fusions. Compared to sg100, the fluorescence of GFP variants such as fr or sf does increase but in split-GFP reassembly assays this comes at the cost of a parallel increase in the background. Consequently, the SNR decreases as the fluorescence increases. In this respect, the worst reporter is sf because it cannot be used even with high-affinity interactions (Figure 6). So, contrary to what might have been expected, fr cannot be used for studying low-affinity interactions because low affinity means the slow reassembly of the bait and the prey compared to the fast reassembly of the two halves of fr. Thus, the study of proteins interacting with a low affinity (i.e., reassembling slowly) seems to be restricted to slowly folding/slowly reassociating/low fluorescence variants. Therefore, only sg100 was selected for further experiments.

### 2.7. Impact of NGFP/CGFP Fusion Inversion

**Figure 12:**
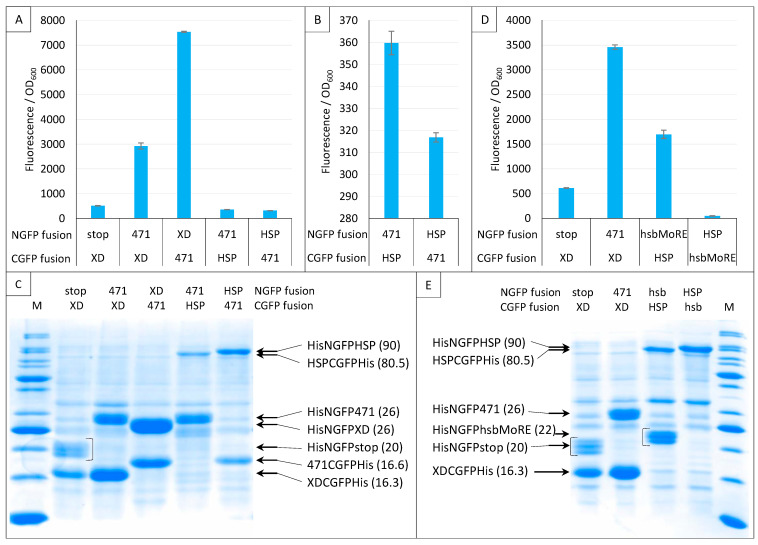
Swapping NGFP and CGFP fusions. Split-GFP reassembly assay results as obtained when swapping the proteins fused to NGFP and CGFP and with the sg100 GFP reporter at 17 °C for 24 h. In this experiment, CGFP fusions were also His-tagged. Fluorescence to OD_600_ ratios obtained with 471 and XD or HSP (**A**), with 471 and HSP (**B**), with hsbMoRE and HSP (**D**) are shown. (**C**) SDS-PAGE analysis of the expression of the NGFP and CGFP fusions of A and B. (**E**) SDS-PAGE analysis of the expression of the NGFP and CGFP fusions of D. M, molecular size markers (200, 150, 100, 85, 70, 60, 50, 40, 30, 25, 20, 15, 10 kDa). In parentheses is indicated the molecular mass in kDa.

Next, we investigated the impact of NGFP/CGFP fusion inversion (Figure 12). Note that these experiments only used sg100, and that a His-tag was also appended to the C-terminal end of CGFP fusions. Both 471/XD and 471/HSP70 pairs were tested. Figure 12A shows that the 471/XD pair provided more fluorescence when XD was fused to NGFP (His-NGFP-XD) and 471 was fused to CGFP (471-CGFP-His) than in the reciprocal combination (His-NGFP-471/XD-CGFP-His). 

Interestingly, the opposite result was obtained for the 471/HSP70 pair (Figure 12A,B): His-NGFP-471/HSP-CGFP-His provided more fluorescence than His-NGFP-HSP/471-CGFP-His, suggesting that the behavior of the same protein (471) toward a given interaction partner (HSP70) cannot be reliably anticipated from its behavior toward another one (XD). A similar observation was also reported by [15] using the frGFP variant. In that case, while the NfrGFP-BARD1/BRCA1-CfrGFP pair yielded a detectable fluorescence, the NfrGFP-BRCA1/BARD1-CfrGFP pair did not. Interestingly, in that study, no fluorescence was detected when sg100 was used instead of fr, whatever the combination of fusions used, suggesting that the choice between different GFP variants might be protein pair dependent. Likewise, ref. [37] also reported that while a combination made of either the helical domain (HD) or the first oligonucleotide/oligosaccharide binding fold (OB1) of BRCA2 fused to NfrGFP is able to interact with the intrinsically disordered peptide DSS1 fused to CfrGFP and to generate fluorescence, the opposite combination (NfrGFP-DSS1/HD-CfrGFP or NfrGFP-DSS1/OB1-CfrGFP) is not.

Figure 12C confirmed the presence of all fusion proteins. However, except for 471/XD, where the amount of the XD fusion mirrors fluorescence values, it is difficult to find a simple relationship linking fluorescence values and the amount of N- or C-GFP fusions.

Considering the low fluorescence provided by the low-affinity 471/HSP70 interaction, we repeated this experiment using an artificial peptide (hsbMoRE) that we previously showed to be able to bind HSP70 with a higher affinity than its native counterpart, i.e., the wild-type MoRE that is responsible for binding to both XD and HSP70 (see Figure 1) [30]. As shown in Figure 12D, similar results were obtained except that, as expected, the fluorescence signal provided by His-NGFP-hsbMoRE/HSP-CGFP-His was about five times higher than that obtained with His-NGFP-471/HSP-CGFP-His. Surprisingly, the fluorescence level obtained when the reciprocal combination was used was close to zero, i.e., a value that was even much lower than that of the negative control His-NGFP-stop/XD-CGFP-His. This extremely low fluorescence value suggests either a lack of expression or a rapid degradation of hsbMoRE-CGFP-His. This assumption was confirmed by the absence of a specific band in the expected migration zone of hsbMoRE-CGFP-His (12.5 kDa, Figure 12E). Although the absence of a band corresponding to hsbMoRE-CGFP-His could in principle reflect either degradation or lack of expression, the latter hypothesis is less likely because hsbMoRE is efficiently expressed and functional when fused to His-NGFP, and CGFP-His is an efficient fusion partner for other proteins (Figure 12C,E).

In conclusion, the experiments reported in Figure 12 show that the choice of an NGFP versus a CGFP fusion has a significant impact on the resulting fluorescence. We thus recommend to (i) generate and assess both NGFP/CGFP combinations and (ii) confirm the presence of both GFP fusions via IMAC purification and SDS-PAGE analysis.

### 2.8. Choice of Negative Controls

The use of negative controls is of course indispensable to enable a meaningful analysis of the results. Accordingly, we always included a negative control in the experiments reported in Figure 1, Figure 2, Figure 3, Figure 4, Figure 5, Figure 6, Figure 7, Figure 8, Figure 9, Figure 10, Figure 11 and Figure 12. Of note, it was the value of the negative control that prompted us to suspect the lack of hsbMoRE-CGFP-His in the experiments reported in Figure 12D, a hypothesis that was confirmed by the gel analysis in Figure 12E.

**Figure 13:**
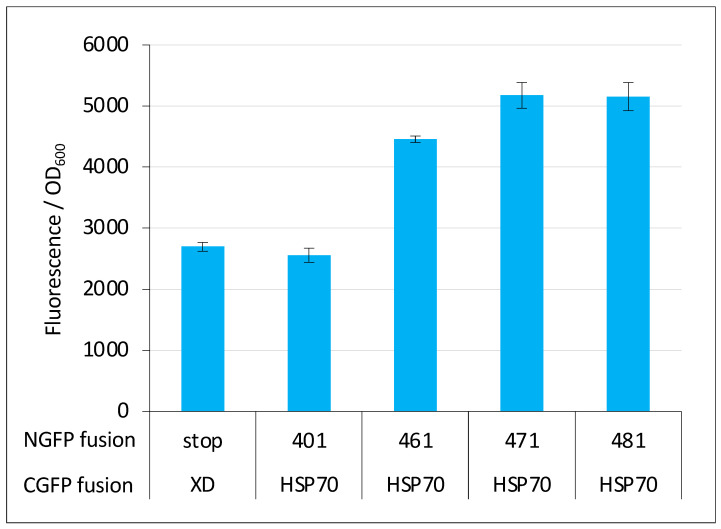
Negative control can be misleading. The fluorescence-to-OD_600_ ratio (mean value and standard deviation of a triplicate experiment) of Ntail/XD pairs and Ntail/HSP pairs indicated on the x-axis is reported. NGFP fusions 461, 471 and 481 are N-terminal truncation variants of full length Ntail (401) starting at residue 461, 471 and 481, respectively, instead of residue 401 for full length Ntail. In these truncation variants, the N-terminal fuzzy region located upstream of the MoRE is progressively reduced. The negative control made of the NGFP (referred to as “stop”)/XD-CGFP pair is used as reference. See Figure 1 for details. Data taken from [29].

However, negative controls should be used with caution in split-GFP reassembly assay because they can lead to false negative results. In our experience, for pairs of small proteins that are both highly expressed in a soluble form in *E. coli* and featuring a relatively high interaction affinity (i.e., K_D_ in the μM range), negative controls are reliable. By contrast, protein pairs endowed with opposite characteristics provide fluorescent signals that can be close enough to, or even lower than that of the negative control (i.e., the fluorescence of bacteria expressing either the NGFP/CGFP pair only or a pair where only one of the two GFP halves is fused to a protein) to be wrongly considered as non-interacting. We experienced such a situation when we first tested the interaction of full-length Ntail (401, see Figure 1A) with HSP70. Although this interaction was known for a long time [25,26,27], our results reproducibly indicated that the Ntail interaction with HSP70 did not provide more fluorescence than the negative control (compare the first two bars in Figure 13). However, the reproducibility of the results and the very small standard deviations suggested that these weak fluorescence signals did in fact account for bona fide interactions. Indeed, truncated variants of Ntail were found to provide fluorescence signals that increased with the truncation (compare the last four bars in Figure 13, adapted from [29]) and prompted us to no longer include a negative control in experiments involving HSP70 or its derivatives. This choice ultimately allowed us to devise a peptide (i.e., hsbMoRE) that binds HSP70 more tightly than its wild-type counterpart ([30] and Figure 12D). Interestingly, hsbMoRE binds HSP70 with an apparent affinity comparable to that of another peptide [38] that was selected for possible therapeutic approaches targeting HSP70.

Another example of aberrant negative control values is provided by the experiments making use of the sf variant where positive and negative controls provided the same fluorescence value (Figure 5).

A plausible explanation for such aberrant negative control values could be the following: a negative control is reliable only when the reassociation reaction is driven by the two proteins of interest (for example 471 and XD). In that case, the reassembly of the two GFP halves is the consequence of this first association, leading to a reliable fluorescence value and to a SNR above 1. Conversely, when the two GFP halves reassociate before the two proteins of interest, which is the case either when the two proteins of interest have a low affinity (471/HSP70) or when the two GFP halves have been evolved to refold robustly (i.e., fr, sf), the fluorescence is no longer a reliable reporter of the binding between the two proteins of interest and the SNR is below 1.

### 2.9. Split-GFP Reassembly Assay and Artificial IDPs

**Figure 14:**
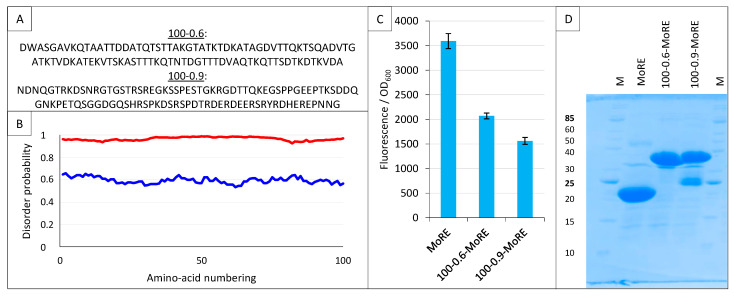
Split-GFP reassembly assay and artificial disordered proteins. (**A**) Amino acid sequence of the two artificial proteins 100-0.6 and 100-0.9. (**B**) Disorder probability of 100-0.6 (blue line) and 100-0.9 (red line), as assessed by IUPred [40]. (**C**) Fluorescence-to-OD_600_ ratio (mean value and standard deviation of a triplicate experiment) of MoRE/XD, 100-0.6-MoRE/XD and 100-0.9-MoRE/XD pairs. (**D**) SDS-PAGE analysis of the expression of the NGFP fusions (His-NGFP-MoRE (24 kDa), His-NGFP-100-0.6MoRE (34 kDa), His-NGFP-100-0.9MoRE (34 kDa)) of C. M, molecular size markers (kDa).

Among different applications, we previously used the split-GFP reassembly assay to validate a software (InSiDDe) that we designed for generating artificial IDPs of given length and disorder probability (http://insidde.afmb.univ-mrs.fr/) [39]. In particular, we evaluated the ability of InSiDDe to generate a sequence that could be effectively expressed in *E. coli*. To this end, we expressed a construct encoding a 100-residue long artificial IDP with a disorder probability of 0.6 (referred to as 100–0.6 in Figure 14A,B), and we tested its binding behavior by split-GFP reassembly [39].

Here, we used InSiDDe to generate another artificial IDP of the same length but with a disorder probability of 0.9 (referred to as 100-0.9 in Figure 14A,B), and we compared the behavior of 100-0.9 to that of the less disordered IDP 100-0.6 that was previously investigated [39]. To this end, the two artificial IDPs were fused upstream of the MoRE of Ntail, i.e., the region encompassing residues 485-506 (Figure 1A) and responsible for binding to XD [23,24]. The resulting constructs are referred to as 100-0.6-MoRE and 100-0.9-MoRE. The sequences coding for these proteins were individually inserted in pNGG to yield His-Nsg100GFP-100-0.6-MoRE and His-Nsg100GFP-100-0.9-MoRE. The ability of His-Nsg100GFP-MoRE, His-Nsg100GFP-100-0.6-MoRE and His-Nsg100GFP-100-0.9-MoRE to interact with XD-Csg100GFP was assessed. As shown in Figure 14C, the MoRE provided the highest interaction, followed by 100-0.6-MoRE and then 100-0.9-MoRE. This finding is in agreement with previous results that showed that the presence of the N-terminal Ntail fuzzy appendage preceding the MoRE dampens the interaction with XD, with this dampening effect being enhanced when the wild-type sequence is replaced with an artificial and more disordered fuzzy sequence that is different from 100-0.6 and 100-0.9, and that was generated without the use of InSiDDe [29]. The results reported in Figure 14C confirm that the inhibitory effect of the Ntail fuzzy appendage increases proportionally to the disorder probability of the fuzzy region. Figure 14D indicates that both fusions containing the artificial disordered appendage are well expressed, although some degradation is observed for the most disordered one.

These results show that the split-GFP reassembly assay is sensitive enough to capture modulation in the binding affinities resulting from a disorder probability shift of the IDP fuzzy appendage as small as 0.3 (i.e., from 0.9 to 0.6).

## 3. Conclusions

The split-GFP reassembly assay devised in Lynne Regan’s lab is particularly well suited for studying IDP interactions. Indeed, we used this assay in the context of a descriptive random mutagenesis approach that targeted Ntail and provided site-resolved information on Ntail region(s) that are critical for binding to XD [28]. We also used this assay to study the effect of the N-terminal fuzzy appendage of Ntail [29] and of varying extents of the helicity of its MoRE on Ntail binding to XD and to HSP70, as well as in the context of an alanine scanning mutagenesis that targeted the MoRE and unveiled that binding to XD and to HSP70 relies on a different set of residues [30]. Finally, we used it to conceive and validate a peptide derived from the MoRE that binds HSP70 with a higher affinity than wt MoRE [30], and to validate an online software we devised to generate in silico artificial IDPs ([39] and this study). We are currently using it to set up a protein-binding competition assay (Bignon C., unpublished results).

The present study unveiled a few critical parameters that deserve consideration when using this powerful approach to investigate PPIs. In particular, the culture medium brand, the expression conditions, the GFP variant used, how interacting proteins are fused to GFP halves, and the affinity of the interacting pair are all parameters that have a considerable impact. In particular, this study revealed the critical importance of having the highest possible binding of protein X to protein Y, to which high affinity, high expression and high solubility contribute, and of the lowest affinity and solubility for rep1 and rep2 to obtain the highest signal and the lowest background, i.e., significant and quantifiable results. Moreover, the results herein presented underscore the importance of quantifying fluorescence and of using affinity-tagged proteins, enabling their expression to be checked by gel electrophoresis.

A better understanding of how the numerous parameters of this technology interplay, to which the present study contributed, holds promise for the future development of an even more sensitive and quantitative assay.

## 4. Materials and Methods

### 4.1. DNA Constructs

All fusion proteins are written from N to C terminal.

#### 4.1.1. Folding Reporter (fr) Constructs

pBS26-frGFP. The folding reporter was constructed according to [14]. Using GFPuv (F99S, M153T, V163A, Q80R) as a template, the folding mutation F64L and the red shift mutation S65T were introduced by overlapping extension PCR using plasmid pGFPuv (Takara Bio USA, Inc. 2560 Orchard Pkwy, San Jose, CA 95131, USA) as a template. PCR1 used primers M13rev and mutGFPuv2, and PCR2 used primers mutGFPuv1 and p17-Amp1. After DpnI treatment, a third PCR used the product of PCR1 and PCR2 as templates and primers M13rev and p17-Amp1. The product of PCR3 was digested with HindIII and SpeI and ligated to pBS26 that had been cut with the same enzymes.

pBS26 is a home-made plasmid derived from pBluescript II phagemid (pBS21) to which five restriction sites (NcoI, NdeI, BglII, MluI, SphI) have been added between EcoRI and PstI (Bignon C., unpublished results). Of note, the fr variant made by [15], 2008 does not contain the Q80R mutation because the fragment coding for residues 1 to 84 was PCR-amplified using as a template the eGFP coding sequence that encodes Q80 and not R80.

pET11a-link-NfrGFP was made by PCR using pBS26-frGFP as a template and primers GFPuv1 and GFPuv2a (an internal XhoI site was mutated by GFPuv2a). After DpnI treatment, the PCR product was digested with NheI and XhoI and then ligated to NheI- and XhoI-digested and gel-purified pET11a-link-Nsg100GFP.

Negative control pET11a-stop-NfrGFP was made by inserting two stop codons downstream of the NGFP coding sequence (hence, the name “Stop” of the construct used throughout the text), followed by a sequence coding for Ntail (see Figure 1A). The reason for keeping the Ntail coding sequence, which is not translated in this construct due to the upstream two stop codons, is to have a negative control as close as possible to the positive control that was full-length Ntail in the first experiments [21]. In practice, the Ntail coding sequence was PCR-amplified with primers XhoIStopNtail and StopNtailBamHI. After DpnI treatment, the PCR product was digested with BamHI and XhoI, and then ligated to BamHI- and XhoI-digested and gel-purified pET11a-link-frNGFP. The translation product of this construct is His-NfrGFP.

Positive control pET11a-471-NfrGFP was made by PCR amplification of the sequence encoding 471 using primers XhoI471 and 471BamHI and pDEST14/NTAILHN as a template [41]. The PCR product was processed as for pET11a-stop-NfrGFP. The translation product of this construct is His-NfrGFP-471.

pNfrGG (pNfrGatewayGFP, the Gateway version of pET11a-link-frNGFP). The Gateway cassette was PCR amplified using pTH31 [42] as a template and overlapping extension PCR to mutate an internal BamHI site [21]. The PCR product was digested with XhoI and BamHI and was ligated to pET11a-link-frNGFP that had been digested with the same enzymes. The construct was not entirely sequenced but its ability to kill CCDB-sensitive cells, in order to confer resistance to chloramphenicol and to be a substrate for LR reaction, was checked.

To generate negative (pNfrGG-stop) and positive (pNfrGG-471) controls, inserts were PCR-amplified using shuttle constructs pDONR-stop and pDONR-471 as templates and Gateway primers attl1a and attL2a [21]. After DpnI treatment, the PCR products were inserted in pNfrGG by LR reaction. The translation products are similar to those expressed by pET11a-stop-NfrGFP and pET11a-471-NfrGFP, except that an attb1-encoded peptide (TSLYKKAGS) is inserted at the end of NGFP. The translation product of these constructs is His-NfrGFP-attb1 and His-NfrGFP-attb1-471, respectively.

pMRBad-XD-CfrGFP was created by overlapping extension PCRs. PCR1 used pMRBad-XD-CspGFP (see further) as a template and primers MluIfrC and AatIIR. PCR2 used pBS26-frGFP as a template and primers GFPuv3a and GFPuv4b. PCR3 used pMRBad-XD-CspGFP as a template and BsrGF and frCHindIII primers. After DpnI treatment, the product of a fourth PCR using PCRs 1 to 3 as megaprimers and primers MluIfrC and frCHindIII was digested with MluI and HindIII and then ligated to HindIII- and MluI-digested pBS26, yielding pBS26-XD-CfrGFP. After sequencing, the insert was subcloned from pBS26 to pMRBad-XD-CspGFP by HindIII and MluI restriction and ligation. The translation product of this construct is XD-CfrGFP.

pMRBad-XD-CfrGFP-His was constructed by overlapping extension PCR. PCRs 1 and 2 used pBS26-XD-CfrGFP as a template and primer pairs GFPuv3a/pMRBadHisR, and pMRBadHisF/T7ter, respectively. After DpnI treatment, a third PCR using PCR 1 and 2 products as a template was run with primers GFPuv3a and T7ter. PCR3 product was digested with AatII et BsrGI, and the 288bp fragment was ligated to pMRBad-XD-CspGFP that had been cut with the same enzymes and gel-purified. The translation product of this construct is XD-CfrGFP-His.

pMRBad-HSP-CfrGFP-His was constructed by digesting pMRBad-XD-CfrGFPHis and pMRBad-HSP-Csg100GFP with MluI and AatII and then ligating the fragments of interest after gel-purification. The translation product of this construct is HSP-CfrGFP-His.

#### 4.1.2. Enhanced GFP (eGFP) Constructs

pET11a-link-NeGFP was constructed by PCR-amplifying the sequence encoding His-NeGFP, using pTH31 as a template [42] and primers NEGFP1 and NEGFP2. After DpnI treatment and NheI and XhoI digestion, the PCR product was ligated to NheI- and XhoI-digested and gel-purified pET11a-link-Nsg100GFP.

pET11a-stop-NeGFP was constructed by ligating the sequence encoding stopNtail (see pET11a-stop-NfrGFP for details) to BamHI- and XhoI-digested pET11a-link-NeGFP. The translation product of this construct is His-NeGFP.

pET11a-471-NeGFP was constructed by ligating the sequence encoding 471 (see pET11a-471-NfrGFP for details) to BamHI- and XhoI-digested pET11a-link-NeGFP. The translation product of this construct is His-NeGFP-471.

pMRBad-XD-CeGFP was constructed, as was pMRBad-XD-CfrGFP, except that PCR2 used pTH31 as a template and primers CEGFP1 + CEGFP2.

#### 4.1.3. Super Positive GFP (sp) Constructs

pET11a-Z-NspGFP and pMRBad-Z-CspGFP encoding leucine zippers fused to His-NGFP and CGFP were obtained from Addgene.

pET11a-stop-NspGFP was constructed as described for pET11a-stop-NfrGFP, except that after BamHI and XhoI digestion the PCR product was ligated to pET11a-Z-NspGFP that had been digested with the same enzymes. The translation product of this construct is His-NspGFP.

pET11a-471-NspGFP was constructed as described for pET11a-471-NfrGFP, except that after BamHI and XhoI digestion the PCR product was ligated to pET11a-Z-NspGFP that had been digested with the same enzymes. The translation product of this construct is His-NspGFP-471.

pMRBad-XD-CspGFP was constructed by PCR-amplifying the XD coding sequence borne by pDEST17 and primers pMR-Nco-M.XD.F and pMR-M.XD-Aat. After DpnI treatment, the PCR product was digested with NcoI and AatII and then ligated with pMRBad-Z-CspGFP that had been digested with the same enzymes and gel-purified. The translation product of this construct is XD-CspGFP.

#### 4.1.4. Super Folder (sf) Constructs

The super folder was derived from the folding reporter (fr) by inserting the following mutations: S30R/Y39N/N105T/Y145F for NGFP and I171V/A206V for CGFP [33].

pET11a-link-NsfGFP was constructed by overlapping extension PCR using pNfrGG as a template in three PCRs to insert the above-mentioned mutations. PCR1 used primers T7prom and sfGFP2, PCR2 used primers sfGFP1 and sfGFP4, and PCR3 used primers sfGFP3 and GFPuv2b. After DpnI treatment, a fourth PCR was performed using products of PCRs 1 to 3 as a template and primers p17-Amp3 and GFPuv2b. After NheI and XhoI digestion, the 517 base pairs fragment was ligated to pET11a-link-Nsg100GFP that had been digested with the same enzymes and gel-purified.

pET11a-stop-NsfGFP was constructed as described for pET11a-stop-NfrGFP, except that after BamHI and XhoI digestion the PCR product was ligated to pET11a-link-NsfGFP that had been digested with the same enzymes. The translation product of this construct is His-NsfGFP.

pET11a-471-NsfGFP was constructed as described for pET11a-471-NfrGFP, except that after BamHI and XhoI digestion the PCR product was ligated to pET11a-link-NsfGFP that had been digested with the same enzymes. The translation product of this construct is His-NsfGFP-471.

pMRBad-XD-CsfGFP was constructed by overlapping extension PCR using pBS26-frGFP as a template and two PCRs to create the above-mentioned mutations. PCR1 used primers CsfGFP1b and sfGFP6, and PCR2 used primers sfGFP5 and GFPuv4b. After DpnI treatment, a third PCR was performed using products of PCRs 1 and 2 as a template and primers CsfGFP1b and GFPuv4b. After digestion with AatII et BsrGI, the PCR product was ligated to pMRBad-XD-CspGFP that had been digested with the same enzymes and gel-purified. The translation product of this construct is XD-CsfGFP.

#### 4.1.5. sg100 Constructs

Constructs using the Gateway version of pET11a-link-Nsg100GFP (i.e., pNGG-stop pNGG-401, pNGG-461, pNGG-471 and pNGG-481) and pMRBad-XD-Csg100GFP have been already described [21,29]. Constructs pNGG-100-0.6-MoRE and pNGG-100-0.9-MoRE have been described in [39]. Plasmids pET11a-Z-Nsg100GFP and pMRBAD-Z-CGFP were kindly provided by Dr Lynne Regan.

pMRBad-HSP-Csg100GFP was constructed by PCR-amplifying the full-length human HSP70 coding sequence (residues 1–641) using pDest14/Hsp72HN as a template [25] and primers Hsp72BspHI and Hsp72AatII. After DpnI treatment, the PCR product was digested with BspHI and AatII and ligated to pMRBAD-link-Csg100GFP that had been digested by the same enzymes. The translation product of this construct is HSP-Csg100GFP.

For making pMRBad-HSP-Csg100GFP-His, pMRBad-XD-Csg100GFP-His and pMRBad-HSP-Csg100GFP were digested with MluI and AatII, and after gel-purification the fragments of interest were ligated. The translation product of this construct is HSP-Csg100GFP-His.

pNGG-XD. The XD coding sequence borne by pDEST14 [23] was transferred to pDONR201 by BP reaction. E. coli cells were transformed with the BP reaction mix and plated on kanamycin plates. The plasmid contained in one colony was purified and used to transfer XD coding sequence to pNGG by LR reaction. The translation product of this construct is His-Nsg100GFP-XD.

pNGG-HSP was constructed by PCR-amplifying the full-length human HSP70 coding sequence (residues 1-641) using pDest14/Hsp72HN as a template [25] and primers attB1-Hsp72 and Hsp72-attB2-AS. After DpnI treatment, the PCR product was inserted in the shuttle vector pDONR201 by BP reaction, and then from pDONR201 to pNGG by LR reaction. The translation product of this construct is His-Nsg100GFP-HSP.

pNGG-hsbMoRE has already been described [30]. The translation product of this construct is His-Nsg100GFP-hsbMoRE.

For constructing pMRBad-hsbMoRE-Csg100GFP-His, the sequence encoding hsbMoRE was PCR-amplified using hsbMoRE-p17Tet as a template (Bignon C., unpublished construct) and primers NcoIhsb and hsbAatII. After DpnI treatment, the PCR product was digested with NcoI and AatII and ligated to the fragment of interest of pMRBad-XD-Csg100GFP-His that had been digested with the same enzymes and gel-purified. The translation product of this construct is hsbMoRE-Csg100GFP-His.

For constructing pMRBad-471-Csg100GFP-His, the sequence coding for 471 was PCR-amplified using pNGG-471 [29] as a template and primers BspHI471 and 471AatII. After DpnI treatment, the PCR product was digested with BspHI and AatII and then ligated to pMRBad-XD-Csg100GFP-His that had been digested with NcoI and AatII. The translation product of this construct is 471-Csg100GFP-His.

Selection and amplification of all the constructs was carried out in *E. coli* TAM1 competent cells (Active Motif, Inc. 1914 Palomar Oaks Way, Suite 150 Carlsbad, CA, USA). The sequence of the coding region of all constructs was checked (GATC (Eurofins Genomics Germany GmbH Anzinger Str. 7a 85560 Ebersberg Germany) or GeneWiz (GeneWiz France LTD, 4 rue de Marivaux, 75002 Paris, France) and found to conform to expectations. The sequences of all the primers used in this study are shown in Supplementary Table S1.

### 4.2. Split-GFP Reassembly Assay

Except for the experiment reported in Figure 9 where a single plasmid was used in some experimental points, T7pRos cells were co-transformed with a plasmid expressing NGFP fusions of the pET11a or the Gateway series and with a plasmid expressing CGFP fusions of the pMRBad series, then grown overnight at 37 °C on ampicillin (100 µg/mL) and kanamycin (50 µg/mL) plates. The next day, colonies from each plate were scraped off and used to seed 4 mL of LB (lysogeny broth) containing the same antibiotics and chloramphenicol (34 µg/mL) in polypropylene 24-well deep-well plates, then incubated overnight at 37 °C under constant shaking. The next day, 4 mL of TB (terrific broth) containing the same three antibiotics were seeded in triplicate with 100 µL of the preculture and incubated for 1.5 h at 37 °C under shaking in 24-well deep-well format. Once this time elapsed, IPTG (0.5 mM) and arabinose (2%) (or either IPTG or arabinose in experiments using only one inducer) were added to each well, and the deep-well was incubated overnight at 17 °C under shaking. The next day, the deep-well plate was spun for 2 min at 2000× *g* and the culture medium was discarded. The cell pellet of each well was resuspended in 1 mL of PBS. Ten microliters of the resuspended pellet were diluted by adding 100 µL of PBS, and the OD_600_ and the fluorescence of this dilution were recorded using a TECAN GENios Plus plate reader (TECAN France, Tour Swiss Life, 1 Bd Marius Vivier Merle, 69003 Lyon, France) and clear bottom, black 96-well plates. Data were processed using Excel. The fluorescence (in arbitrary units) was divided by the OD_600_ and the mean and standard deviation of the ratio of each triplicate was calculated.

### 4.3. Analysis of Protein Expression

The same OD_600_, generally comprised between 10 and 50, was used for all experimental points of a given experiment. The cells were spun for 2 min at 2000× *g* and the supernatant discarded. The cell pellet was resuspended in 1 mL of lysis buffer (50 mM of Tris pH8, 300 mM of NaCl, 10 mM of imidazole, 1 mM of EDTA, 0.1% Triton X100, 0.25 mg/mL of lysozyme, 1 mM of PMSF) and frozen. After thawing, the cell lysate was supplemented with 20 mM of MgSO4 and 20 µg/mL of DNAse I and incubated at 37 °C for 30 min under shaking. Each lysate was supplemented with urea to 8 M and incubated under the same conditions for an additional 30 min. Each denatured lysate was transferred to 2 mL tubes that were supplemented with 100 µL of a 50% (volume/volume) suspension of IMAC Sepharose high performance (Cytiva, 24 avenue de l’Europe 78140 Vélizy-Villacoublay, France) and incubated at room temperature for 30 min on a rotating wheel. The tubes were then centrifuged for a few seconds at 2000× g and the supernatant was discarded. The beads were resuspended in 1 mL of 50 mM Tris pH8, 300 mM NaCl and 8 M urea and spun again. After discarding the supernatant, the beads were resuspended in 200 µL of 50 mM Tris pH8, 300 mM NaCl, 8 M urea and 250 mM imidazole, and 10 µL of the mixture containing the beads was withdrawn immediately after having shaken the tube and subsequently analyzed by SDS-PAGE.

## Data Availability

The data present in the current study are available from the corresponding authors on reasonable request.

## Supplementary Materials

The following are available online at https://www.mdpi.com/article/10.3390/ijms232113167/s1.
