# Supplementary material for: Split-GFP Reassembly Assay: Strengths and Caveats from a Multiparametric Analysis
Source: Int J Mol Sci. 2022 Oct 29;23(21):13167. doi: 10.3390/ijms232113167 (PMC9658207; doi:10.3390/ijms232113167)
Supplement: Supplementary file 1 [file ijms-23-13167-s001.zip › ijms-1968998-supplementary.pdf]

Supplementary Information

Split-GFP reassembly assay: strengths and caveats from a multiparametric analysis

Christophe Bignon[1*], Antoine Gruet[1#] and Sonia Longhi[1*]

[1]Architecture et Fonction des Macromolécules Biologiques (AFMB), Centre National de la Recherche Scientifique (CNRS), Aix-Marseille Université (AMU), UMR 7257, 13288 Marseille, France
[#] present address: Cold Spring Harbor Laboratory, Cold Spring Harbor, NY 11724, USA

*to whom correspondence should be sent

Christophe Bignon
AFMB, UMR 7257 CNRS and Aix-Marseille University
163, avenue de Luminy, Case 932, 13288 Marseille Cedex 09, France
Tel: (33) 4 91 82 55 80; Fax: (33) 4 91 26 67 20
E-mail: christophe.bignon@univ-amu.fr

Sonia Longhi
AFMB, UMR 7257 CNRS and Aix-Marseille University
163, avenue de Luminy, Case 932, 13288 Marseille Cedex 09, France
Tel: (33) 4 91 82 55 80; Fax: (33) 4 91 26 67 20
E-mail: sonia.longhi@univ-amu.fr

Supplementary Figures S1
Supplementary Table S1

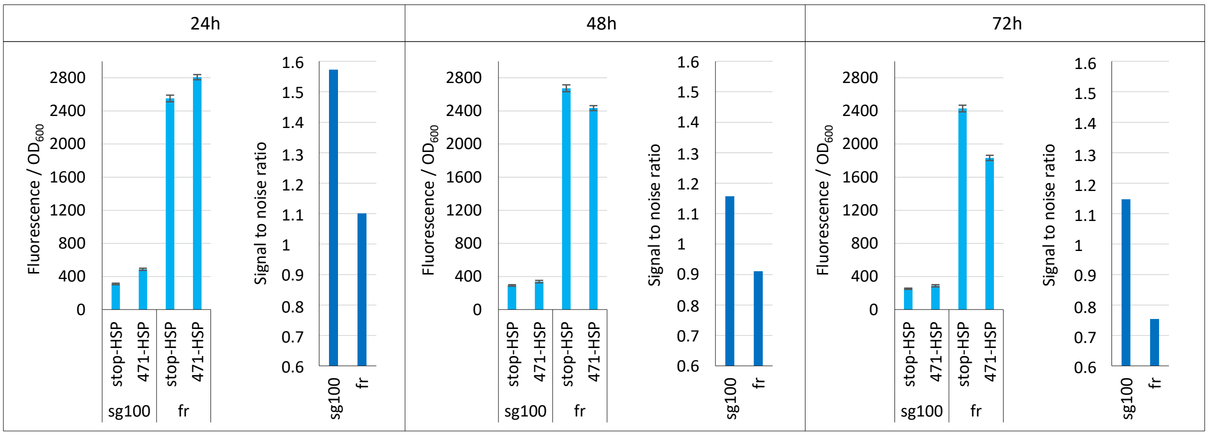

Supplementary Figure S1. Fluorescence to OD_{600} ratios and signal to noise ratio of a split-GFP reassembly assay comparing results obtained at 24h, 48h and 72h with sg100 and fr and with stop-HSP and 471-HSP at 17°C.

Supplementary Table S1. Sequence of the primers used in this study.

Primer	Sequence
mutGFPuv1	GTCACTACTCTGACCTATGGTGTTCAATGCTT
mutGFPuv2	CCATAGGTCAGAGTAGTGACAAGTGTTG
p17-Amp1	GGGGTTCCGCGCACATTTCC
GFPuv1	GATATACATATGGCTAGCCATCACCACCATCACCATGGCGCGAGTAAAGGAGAAGAACTTTTC
GFPuv2a	ACCTTGTTTGTCTTCCCGGGATTGCTCGAGCCAGAGCCAGAGCCTGCCGTGATGTATACATTGTGTGAGTTATAGTTGTACTCCAGTTTG
MluIfrC	GCCATGACAAAAACGCGTAACAAAAGTGTCTATAATCAC
AatIIR	GACGTCGACTTCATTATTAT
GFPuv3a	ATAATAATGAAGTCGACGTCGGGTGGAAGCGGTAAGAATGGAATCAAAGCTAAC
GFPuv4b	GCCGGATCCTTAGTTGTACATCATTATTTGTAGAGCTCATC
BsrGF	TGTACAACTAAGGATCCGGC
frCHindIII	AAAGAAATGCATAAGCTTTTGCCATTCTCACCGGATTC
XhoIStopNtail	CTCTGGCTCTGGCTCGAGCTAATAAACTACTGAGGACAAG
StopNtailBamHI	TTGTTAGCAGCCGGATCCTTATTAGTCTAGAAGATTTC
XhoI471	CTCTGGCTCTGGCTCGAGCACACCCCTAGACATTGACAC
471BamHI	TTGTTAGCAGCCGGATCCTTATTAGTCTAGAAGATTTCTGTCATTG
sfGFP1	AGAGGGTGAAGGTGATGCAACAAACGGAAAAC
sfGFP2	TTGCATCACCTTCACCCTCTCCACGGACAGAAAATTT
sfGFP3	GATGACGGGACCTACAAGAC
sfGFP4	GTCTTGTAGGTCCCGTCATC
GFPuv2b	TTCCCGGGATTGCTCGAGCCAGAGCCAGAGCCACCTTGTTTGTCTGCCGTGATGTATACATTGTGTGAGTTAAAGTTGTAC
p17-Amp3	GATGGTGTCCGGGATCTCGACGC
CsfGFP1b	ATAATAATGAAGTCGACGTCGGGTGGAAGCGGTAAGAATGGA ATCAAAGCTAACTTCAAAATTCGCCACAACGTGGAAGATGG
GFPuv4b	GCCGGATCCTTAGTTGTACATCATTATTTGTAGAGCTCATC
sfGFP5	GACACAATCTGTGCTTTCGAAAG
sfGFP6	CTTTCGAAAGCACAGATTGTGTC
GFPuv3a	ATAATAATGAAGTCGACGTCGGGTGGAAGCGGTAAGAATGGAATCAAAGCTAAC
pMRBadHisR	ATGGTGATGGTGGTGATGTTTGTAGAGCTCATCCATGC
pMRBadHisF	CATCACCACCATCACCATTAATGATGTACAACTAAGGAT
attB1-Hsp72	GGGGACAAGTTTGTACAAAAAAGCAGGCTCTGCCAAAGCCGCGGCAGTCGG
Hsp72-AttB2-AS	GGGGACCACTTTGTACAAGAAAGCTGGGTCTTAATCTACCTCCTCAATGGTGGGGCC
Hsp72BspHI	AAGGAGATATACTCATGAGCGCCAAAGCCGCGGCAGTCGG
Hsp72AatII	CCGCTTCCACCCGACGTCGAATCTACCTCCTCAATGGTGGGGCC
BspHI471	AAGGAGATATACTCATGAGCACACCCCTAGACATTGACAC
471AatII	CCGCTTCCACCCGACGTCGAGTCTAGAAGATTTCTGTCATTG

NeGFP1	GATATACATATGGCTAGCCATCACCACCATCACCATATGGTGAGCAAGGGCGAGGAG
NeGFP2	TTCCCGGGATTGCTCGAGCCAGAGCCAGAGCCACCCTGCTTGTCGGCCATGATATAG
CEGFP1	ATAATAATGAAGTCGACGTCGGGTGGAAGCGGTAAGAACGGCATCAAGGTGAAC
CEGFP2	GCCGGATCCTTAGTTGTACATCATTACTTGTACAGCTCGTCCATGCC
XhoIStopNtail	CTCTGGCTCTGGCTCGAGCTAATAAACTACTGAGGACAAG
StopNtailBamHI	TTGTTAGCAGCCGGATCCTTATTAGTCTAGAAGATTTC
XhoI471	CTCTGGCTCTGGCTCGAGCACACCCCTAGACATTGACAC
471BamHI	TTGTTAGCAGCCGGATCCTTATTAGTCTAGAAGATTTCTGTCATTG
pMR-Nco-M.XD.F	CCATG GGC GCA TCA CGC AGT GTA ATC CGC TCC
pMR-M.XD-Aat	GACGTCGACTTCATTATTATCTTCACCAGCAT
sfGFP1	AGAGGGTGAAGGTGATGCAACAAACGGAAAAC
sfGFP2	TTGCATCACCTTCACCCTCTCCACGGACAGAAAATTT
sfGFP3	GATGACGGGACCTACAAGAC
sfGFP4	GTCTTGTAGGTCCCGTCATC
GFPuv2b	TTCCCGGGATTGCTCGAGCCAGAGCCAGAGCCACCTTGTTTGTCTGCCGTGATGTATACATTGTGTGAGTTAAAGTTGTAC
p17-Amp3	GATGGTGTCCGGGATCTC GACGC
CsfGFP1b	ATAATAATGAAGTCGACGTCGGGTGGAAGCGGTAAGAATGGA ATCAAAGCTAACTTCAAAATTCGCCACAACGTGGAAGATGG
sfGFP5	GACACAATCTGTGCTTTCGAAAG
sfGFP6	CTTTCGAAAGCACAGATTGTGTC
NcoIhsb	AAGGAGATATACCCATGGGCCCGCAGGCGGCAGCCGCGAG
hsbAatII	CCGCTTCCACCCGACGTCGATTCCGACGCGGCGCCCGCGC
XhoIStopNtail	CTCTGGCTCTGGCTCGAGCTAATAAACTACTGAGGACAAG
StopNtailBamHI	TTGTTAGCAGCCGGATCCTTATTAGTCTAGAAGATTTC
XhoI471	CTCTGGCTCTGGCTCGAGCACACCCCTAGACATTGACAC
471BamHI	TTGTTAGCAGCCGGATCCTTATTAGTCTAGAAGATTTCTGTCATTG
attL1a	ACCTGTTCGTTGCAACAAATTGATGAGCAATGCTTTTTTATAATGCCAAGTTTGTACAAAAAAGCAGGC
attL2a	TTTTGACTGATAGTGACCTGTTCGTTGCAACAAATTGATAAGCAATGCTTTCTTATAATGCCCACTTTGTACAAGAAAGCTGGG
